# Structural Impact of 4‐Hydroxynonenal Modification on Human Cytochrome CYP4F11

**DOI:** 10.1002/cmdc.202500935

**Published:** 2026-01-31

**Authors:** Olena Gnatyuk, Oleksii Skorokhod, Alessandro Damin, Mykhailo Chaika, Fateme Naeimaeirouhani, Loris Pica, Anita Tomatis, Aleksandra Smorygo, Taras Voitsitskyi, Gianluca Catucci, Galyna Dovbeshko, Gianfranco Gilardi

**Affiliations:** ^1^ Institute of Physics National Academy of Sciences of Ukraine Kyiv Ukraine; ^2^ Department of Life Sciences and Systems Biology University of Torino Torino Italy; ^3^ Department of Chemistry University of Torino Torino Italy; ^4^ Institute of Low Temperatures and Structural Research PAN Wroclaw Poland; ^5^ Receptor.AI Inc. London UK

**Keywords:** 4‐hydroxynonenal (4‐hNE), CYP4F11, differential scanning calorimetry, Fourier transform infrared (FTIR) spectroscopy, lipid peroxidation, luminescence, P450, post‐translational protein modifications, raman spectroscopy

## Abstract

Posttranslational modifications of human enzymes play a crucial role in disease development. 4‐hydroxynonenal (4‐HNE), a lipid peroxidation product, can modify proteins and disrupt their function. Human cytochrome CYP4F11, involved in lipid metabolism and xenobiotic degradation, was previously shown to be inhibited by 4‐HNE in a malaria model, where hemozoin‐induced 4‐HNE formation occurs in monocytes. However, structural changes to CYP4F11 upon 4‐HNE modification had not been described. In this study, we investigated these changes using differential scanning calorimetry (DSC), Fourier transform infrared (FTIR), and Raman spectroscopy. DSC thermograms revealed an increased energetic barrier to unfolding, suggesting structural reorganization. FTIR data, supported by computational analysis, showed a decrease in alpha‐helix content (0.2–2.5%) and an increase in beta‐structure (2.2–3.3%), along with altered disordered regions. Raman spectroscopy indicated significant changes in luminescence decay across emission wavelengths. These structural alterations induced by 4‐HNE conjugation (protein lipoxidation) may significantly influence the enzymatic activity of CYP4F11, with potential implications for lipid metabolism and xenobiotic detoxification.

## Graphical abstract

Protein lipoxidation by 4‐hydroxynonenal induces conformational and stability changes in human CYP4F11, as revealed by DSC, FTIR, and Raman spectroscopy, with potential consequences for enzymatic function.

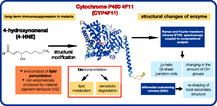

## 1 Introduction

Numerous enzyme modifications, including post‐translational modifications (PTMs), are inherent components of biochemical processes [1, 2, 3]. Moreover, certain modifications have been described as crucial features of various diseases. These modifications can either be the result of disease development [4, 5], or itself be the cause of the pathology [6, 7], or both [5, 8]. For example, malaria is characterized by elevated oxidative stress and lipoperoxidation [9, 10, 11, 12], as observed in numerous studies in all levels of place of action ranging from single parasites, to parasitized red blood cells and entire organisms [9, 13, 14, 15]. In clinical settings, the presence of pro‐oxidants [16, 17], advanced oxidation protein products, and lipoperoxidation [18, 19, 20] have been detected in the blood of malaria patients. Increased levels of the lipoperoxidation product malondialdehyde during malaria have also been analysed and reviewed [21, 22].

4‐hydroxynonenal (4‐HNE), a terminal product of lipid peroxidation chain reactions, is capable of inducing PTMs [8, 23, 24]. The 4‐HNE is known for its reactivity with proteins and nucleic acids, both in close proximity to the 4‐HNE production site and in relatively distant cells and tissues [4, 9, 25, 26]. Malaria is a disease where the impact of 4‐HNE on pathophysiology is well‐established [9, 27, 28]. The 4‐HNE produced by the malarial pigment hemozoin is capable of modifying cytoskeletal proteins [29], the granulocyte‐macrophage colony‐stimulating factor receptor [30], and the CYP4F11 enzyme in human primary monocytes [31], contributing to long‐term immunosuppression in malaria [32, 33, 34, 35].

Cytochrome P450 enzymes are important for a wide spectrum of metabolic processes, primarily known for their crucial role in the metabolism of drugs and endobiotics [36, 37, 38, 39]. They are involved in, or have an impact on, numerous diseases, extending from liver cirrhosis to infectious diseases such as malaria [31, 38, 40, 41, 42, 43, 44]. The CYP4 family of enzymes is a group of cytochrome P450 enzymes involved in the metabolism of various endogenous and exogenous compounds, including fatty acids, prostaglandins, and xenobiotics [45, 46, 47]. They play critical roles in processes such as lipid metabolism, hormone synthesis, and detoxification [48, 49, 50]. CYP4F11 has attracted increasing interest in the field of oncology [47, 48], and its role in immune regulation has recently gained attention [31]. Nevertheless, several biochemical and biophysical aspects of this enzyme remain unclear, and comprehensive biophysical studies on CYP4F11 are largely lacking. Of particular interest are the PTMs of CYP4F11 by 4‐HNE at specific amino acid residues, which were recently identified and may contribute to enzyme inhibition through mechanisms that are not yet fully understood [31]. Moreover, while the lipid‐rich environment of the endoplasmic reticulum and interactions with cytochrome P450 reductase (CPR) may theoretically influence CYP4F11 function and dysfunction, these complex systems also warrant future investigation. To accurately dissect the molecular details of 4‐HNE‐induced modifications, we considered it essential to first conduct a focused study on the isolated CYP4F11 protein under defined conditions.

Fourier transform infrared (FTIR) and Raman spectroscopy are highly important methods for the sensitive characterization of the secondary structure of proteins. These techniques can reveal even negligible changes in structure and amino acid content, redistribution of intra‐ and intermolecular hydrogen bonds, protein interactions with ligands, and more [51, 52, 53]. Differential scanning calorimetry (DSC) is another powerful technique for studying the structural conformation of proteins. It can measure key parameters such as the thermal transition temperature (melting temperature, *T*_m_) and the energy required to disrupt the interactions stabilizing the tertiary structure (enthalpy, Δ*H*), which are often impacted by ligand binding and PTMs [38, 54, 55, 56].

The goal of this study is to investigate the structural modifications of the human CYP4F11 enzyme induced by 4‐HNE, a reactive lipid peroxidation product implicated in the pathophysiology of malaria and other diseases. Using a combination of spectroscopic techniques (fluorescence, FTIR, Raman) and DSC, along with computational modeling, we aim to elucidate the molecular mechanisms underlying 4‐HNE‐induced alterations in CYP4F11.

## 2 Experimental Section

### 2.1 Expression and Purification of CYP4F11

CYP4F11 was cloned into pCWori (+) vector for heterologous expression in the *E. coli* BL21 strain. The N‐terminal domain of native CYP4F11 was modified by replacing the first 10 amino acids of the wild‐type sequence (MPQLSLSWLG) with a sequence of 8 residues (MALLLAVF), which is widely recommended to enhance the expression of human CYP enzymes in *E. coli* [31, 45, 57, 58], and 4xHis‐tag was introduced at the C‐terminal end. To support heme biosynthesis, 0.5 mM of δ‐aminolevulinic acid (δ‐ALA) was added, and protein expression was induced with 1 mM isopropyl *β*‐D‐1‐thiogalactopyranoside (IPTG). The enzyme was purified using a medium composed of 20 mM potassium phosphate buffer (pH 7.4), 20% glycerol, 0.1% IGEPAL (octylphenoxy poly(ethyleneoxy)ethanol), and phosphate inhibitors. Cell lysis was performed on ice using lysozyme at a concentration of 0.1 mg/mL, followed by sonication consisting of 10 bursts of 20 s each, with 1‐minute cooling periods between bursts. Purification was carried out using a combination of ion exchange chromatography and nickel ion affinity chromatography, with elution performed using a histidine gradient ranging from 1 to 40 mM. Subsequently, the purified protein was concentrated using ultrafiltration with a 30 kDa molecular weight cut‐off membrane (Amicon, Millipore, Burlington, MA, USA) and was buffer‐exchanged to a solution containing 100 mM potassium phosphate at pH 7.4, 20% glycerol, and 500 mM NaCl [31]. The enzyme was then stored at −20°C. Prior to use, the enzyme was thawed, and the storage buffer was replaced via ultrafiltration with a 100 mM potassium phosphate buffer at pH 7.4. The enzyme purity was verified by sodium dodecyl sulphate‐polyacrylamide gel electrophoresis (SDS‐PAGE), while its functionality was confirmed using a carbon monoxide (CO) binding spectral assay, an NADPH consumption assay, and an activity assay toward the substrate 15‐HETE [31, 59, 60].

### 2.2 CO‐Binding Spectral Assay

To verify the proper folding of the purified cytochrome P450, CO‐binding spectral assay was conducted at 25°C. The oxidized CYP4F11 was reduced with sodium dithionite, and its spectrum was measured using an 89090A UV‐VIS spectrophotometer (Agilent, Santa Clara, CA, USA). Subsequently, carbon monoxide was bubbled through the enzyme solution for 30 s, and the spectrum of the resulting reduced carbon monoxide‐bound enzyme form was recorded. The difference between the CO‐bound and reduced spectra was utilized to determine the enzyme concentration, employing the absorbance value at 450 nm and a molar extinction coefficient (*ε*450 nm) of 91 000 M^−1^ cm^−1^ [59, 61].

### 2.3 NADPH Consumption Assay

NADPH consumption in enzymatic reaction was tracked by observing the decrease in NADPH absorbance at 340 nm [60]. The reaction mixture included 5 µM of the CYP4F11 enzyme, 15 µM of human CPR, 500 µM of NADPH, and 150 µM of the substrate 15‐HETE in a 0.1 M potassium phosphate buffer at pH 7.4. The reaction progress was monitored during 800 s at 25°C.

### 2.4 CYP4F11 Enzymatic Activity Assay

The enzymatic activity of CYP4F11 was measured using 15‐HETE as the substrate. The reaction mixture contained 5 µM CYP4F11, 15 µM human CPR, 500 µM NADPH, and 100 µM 15‐HETE in 0.1 M potassium phosphate buffer (pH 7.4). Reactions were incubated for 30 min at 37°C. The reaction was terminated by adding ice‐cold acetonitrile (3 volumes) to rapidly denature and precipitate proteins. The decrease in substrate and formation of product were quantified by high‐performance liquid chromatography (HPLC) (see below), and Kcat values were calculated from three independent CYP4F11 purifications.

### 2.5 CYP4F11 Modification by 4‐HNE

The recombinant CYP4F11 (3 µM) was incubated with 100 µM of 4‐HNE (Cayman, Ann Arbor, Michigan, USA) in a 100 mM phosphate buffer at pH 7.4. The incubation was carried out at 25°C for 15 min in a 100 µL volume and the decrease in free 4‐HNE concentration was monitored by HPLC analysis. Subsequently, any excess 4‐HNE was eliminated through ultrafiltration using ultrafiltration devices (Amicon), and the modified CYP4F11 was concentrated up to necessary concentrations for following analysis. Additionally, CYP4F11 modifications by 4‐HNE were confirmed by western blot (see below).

### 2.6 HPLC Analysis

HPLC analysis of 4‐HNE [62] and separately 15‐HETE and 15,20‐dihydroxyeicosatetraenoic acid (15,20‐diHETE) was performed on an Agilent 1200 Series HPLC system (Agilent Technologies, Santa Clara, CA), equipped with a diode array detector (DAD) and a reverse‐phase column (Zorbax Eclipse Plus C18, 250 × 4.6 mm, 5 μm). A sample volume of 10 μL was injected, and the analysis was conducted at a controlled column temperature of +25°C. For the detection of free 4‐HNE, samples were dissolved in methanol and analysed using a mobile phase consisting of water and acetonitrile (20:80, vol/vol). The chromatographic separation was performed at a constant flow rate of 1 mL/min, with detection at a wavelength of 234 nm. Under these conditions, 4‐HNE was identified with a retention time of 7.69 min. The limit of detection was defined as the lowest signal significantly above background noise, based on five replicate measurements. For the detection of 15‐HETE and 15,20‐diHETE, samples were dissolved in N‐hexane containing 1% (vol/vol) acetic acid and analysed using a mobile phase of N‐hexane/2‐propanol/acetic acid (100:2:0.1, vol/vol/vol). Chromatographic separation was performed at a constant flow rate of 1 mL/min, with detection at a wavelength of 235 nm. Under these conditions, 15‐HETE and 15,20‐diHETE were identified with retention times of 12.53 and 9.87 min, respectively.

### 2.7 CYP4F11 Modification by 4‐HNE Analysis by SDS‐PAGE/WB

CYP4F11 modified and not by 4‐HNE were quantified by Bradford Protein Assay (Merck) and solubilised at 95°C by 5 min incubation in Laemmli buffer, prepared as a 2x concentrated solution containing 125 mM Tris‐HCl at pH 6.8, 4% SDS (wt/v), 20% glycerol (v/v), 5% *β*‐mercaptoethanol (v/v), and 0.01% bromophenol blue (v/v). 1 μg of CYP4F11 was separated with an 8% acrylamide (w/v) SDS‐PAGE and transferred onto a PVDF membrane by western blotting (WB). The membrane was blocked by phosphate‐buffered saline (PBS) supplemented with 2% bovine serum albumin (PBS‐BSA) and incubated for 1 h at room temperature (RT) with the monoclonal primary anti‐4‐HNE‐conjugate antibody developed and gently provided by Koji Uchida o purchased (Abcam, UK; 1:1000 dilution) [30, 63]. Then, the membrane was washed with PBS‐BSA and incubated for 1 h at RT with the secondary HRP‐conjugated antimouse antibody (Pierce, Walhem, MA, USA) at the dilution 1:10000. Positive bands were visualized by enhanced chemiluminescence (ECL) and digitalized with ChemiDoc MP Imaging System and ImageLab 6.1 software (both Bio‐Rad) [31]. Human serum albumin (HSA), in vitro conjugated with 4‐HNE was included as positive control [64, 65]. Shortly, 1µM of HSA was dissolved in 100 mM sodium acetate at pH 4.5 and incubated with 1 mM of 4‐HNE at 37C for 30 min. Eventual nonbound excess of 4‐HNE was removed by centrifuge filtration device (Amicon, Millipore) and 0.5 µg of 4‐HNE‐modified HSA in Laemmli buffer was used as a positive control reference sample in WB.

### 2.8 Raman Spectroscopy Analysis

Raman spectroscopy was employed to investigate structural features of the protein samples. A 244 nm laser line (Coherent 300C MotoFred) and an inVia Raman Microscope (Renishaw, Wotton‐under‐Edge, UK) were used for the measurements. The laser was focused on the sample contained in a quartz capillary using a 15× NUV objective. Rayleigh scattering was eliminated using an edge filter. Protein concentration in the samples was 100 µM.

### 2.9 FTIR Analysis

Fourier‐transform infrared (FTIR) spectroscopy was used to analyze structural changes in CYP4F11 and its 4‐HNE‐modified form. Infrared spectra were recorded using the INVENIO‐R instrument (Bruker, Billerica, Massachusetts, US) in the 3900–600 cm^−1^ range, operating in reflectance mode with a Bio‐ATR (attenuated total reflection) attachment. The wavenumber accuracy was ≈0.01 cm^−1^, and absorbance accuracy was about 0.1%. Samples were deposited onto the working surface of the Bio‐ATR attachment and dried under a nitrogen flow at RT to preserve the protein's structural state at the time of measurement. All FTIR spectra were baseline‐corrected, and absorbance band positions were determined using OPUS 8.2 software (Bruker). Spectral fitting of the FTIR data was performed using the PeakFit 4.0 software. Specific absorbance bands were assigned to distinct structural components of the protein. The alpha‐helical phase was identified in the range of 1651–1652 cm^−1^. Beta‐sheet structures were observed at 1631–1633 cm^−1^, while beta‐turns appeared at 1675–1676 cm^−1^. Disordered regions, corresponding to random coil structures, were detected between 1656–1658 cm^−1^. Side‐chain vibrations were assigned to bands in the ranges of 1608–1619 cm^−1^ and 1700–1710 cm^−1^.

### 2.10 Fluorescence Lifetime Measurement

Time‐resolved fluorescence spectroscopy is a powerful technique widely used in biochemistry, materials science, environmental monitoring, and medical diagnostics due to its ability to provide detailed insights into molecular dynamics (MD), interactions, and structural changes [66, 67]. In this study, excitation and emission spectra were recorded using an FLS1000 fluorescence spectrometer (Edinburgh Instruments, Livingston, UK), equipped with a 450 W ozone‐free xenon lamp as the excitation source. Luminescence decay curves were measured at RT using the same spectrometer, fitted with 258 nm and 370 nm electronic pulsed laser picosecond pulsed diode lasers.

### 2.11 Differential Scanning Calorimetry

The thermal stability of CYP4F11 was evaluated using DSC. Measurements were performed with a Microcal VP‐DSC instrument (Malvern, UK), applying a temperature gradient from 25°C to 80°C at a scan rate of 60°C/h, following a 10 min prescan equilibration period [38, 68, 69]. Thermograms were recorded for both native and 4‐HNE‐modified CYP4F11. To assess dose‐dependence, additional DSC measurements were conducted using CYP4F11 modified with an elevated concentration of 1 mM 4‐HNE. All samples were prepared at a protein concentration of 0.6 mg/mL. Data analysis was carried out using Microcal Origin 6.0 software (Malvern, UK).

### 2.12 Computational Modelling

The predicted structure of the CYP4F11 protein, generated by AlphaFold [70], was utilized in this study. The structure was obtained from the free version of the AlphaFold Protein Structure Database (alphafold.ebi.ac.uk; accessed on 24 February 2024). Specific residues (C45, C260, H261, H347, C354, and K451) were manually modified by Michael addition with 9‐carbon 4‐HNE. Targeted residues were known from our previous study [31]. Subsequently, the modified structure was preprocessed and energy‐minimized using the universal force field (UFF) [71], with the aid of the open‐source cheminformatics software RDKit v.2023.09.1. Before the minimization, the protein was virtually protonated and the force field was obtained using default parameters but nonbonded terms between fragments added to the force field. The minimization was run up to 100 iterations with default parameters. The unmodified CYP4F11 AlphaFold structure was also minimized for fair comparison. The secondary structure fractions were calculated using the dictionary of protein secondary structure (DSSP) v. 4.0.4 [72, 73].

## 3 Results

### 3.1 4‐HNE Conjugation with CYP4F11

In order to test the structural changes induced by the conjugation of CYP4F11 with 4‐HNE, the enzyme was heterologously expressed in *E. coli* and purified [31]. The yield of the purified enzyme was 5 mg/L of bacterial culture, and its purity was confirmed by SDS‐PAGE. The absorbance spectrum of the purified enzyme showed a typical Soret band at 418 nm. To ensure the quality of heterologously expressed and purified CYP4F11, spectral analysis was performed. As expected, CYP4F11 in its oxidized form exhibited a peak at 418 nm, while the reduced CO‐bound form showed a characteristic peak at 450 nm (Figure S1). The correct activity of enzyme was confirmed by NADPH consumption assay with 15‐hydroxyeicosatetraenoic acid (15‐HETE) as CYP4F11 substrate.

**FIGURE 1:**
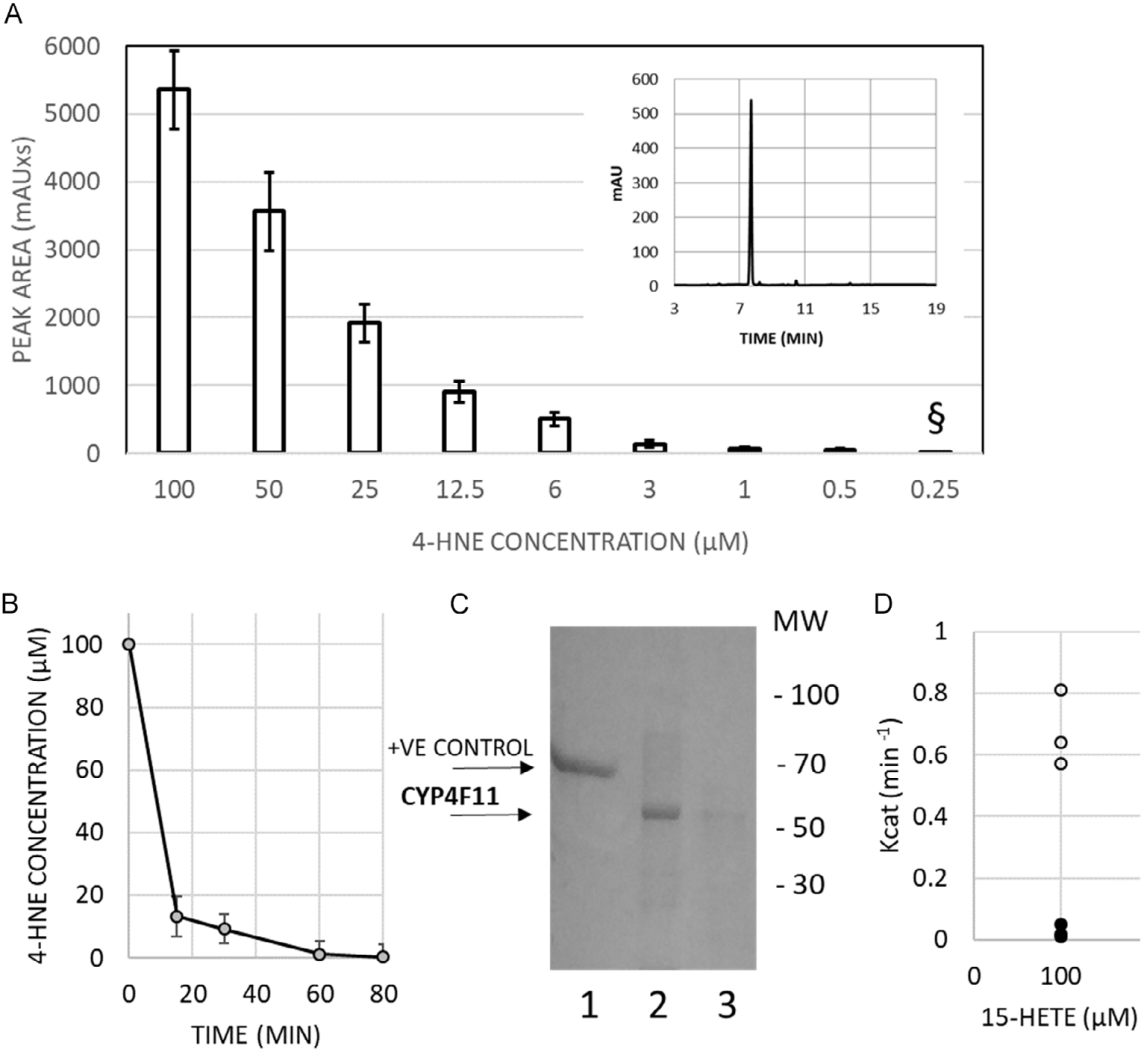
The binding of 4‐HNE to CYP4F11 measured by HPLC and WB. (A) The peak area for 4‐HNE detected by HPLC versus 4‐HNE concentration is plotted as the mean and standard error (SE, *n* = 5 independent preparations). § indicates the detection limit for 4‐HNE by this method. A typical 4‐HNE (25 µM) chromatogram is shown in the inset. (B) 4‐HNE detected in the medium during the binding to CYP4F11. Means and SE are plotted (*n* = 3 independent enzyme purifications). (C) 4‐HNE conjugates with CYP4F11 detected by WB. Lane 1: HSA modified by 4‐HNE used as a positive control (+VE CONTROL); lane 2: CYP4F11 modified by 4‐HNE; lane 3: CYP4F11 unmodified by 4‐HNE used as a negative control; the position of molecular weight (MW) markers is indicated in the right. (D) Catalytic activity of CYP4F11 (open circles) and 4‐HNE–modified CYP4F11 (filled black circles) toward its substrate 15‐HETE (100 µM), expressed as Kcat. Data represent three independent experiments using separate CYP4F11 purifications.

For enzyme modification by 4‐HNE, the purified CYP4F11 was incubated with 4‐HNE at concentrations of 100 µM and 1 mM. Note that the 100 µM concentration of 4‐HNE represents the physiological or pathophysiological level that could be reached in cellular compartments, while the 1 mM concentration is a relatively high experimental level of 4‐HNE. The binding of 4‐HNE to CYP4F11 was monitored using HPLC, which revealed a detection limit for free 4‐HNE of 0.25 µM (Figure 1A). After the initial addition of 100 µM 4‐HNE to the incubation medium with CYP4F11, a rapid decrease in the free 4‐HNE level was observed, indicating 4‐HNE binding to the enzyme. After 15 min of incubation, 87 ± 6% of 4‐HNE was bound (Figure 1B), and 91 ± 5% was bound after 30 min. Binding experiments were performed three times with enzyme samples from three different protein purifications. To confirm the conjugation with 4‐HNE, Western Blotting (WB) analysis was performed to detect 4‐HNE‐protein conjugates with CYP4F11. Specific antibodies were used after protein transfer to a PVDF membrane (Figure 1C). The modification of the enzyme by 4‐HNE following incubation with 100 µM 4‐HNE is shown in Figure 1C, lane 2, as a strong band. The positive control, HSA separately modified by 4‐HNE, appears as a prominent band at 66 kDa in lane 1. In contrast, the negative control—CYP4F11 without 4‐HNE modification—shows no significant signal in lane 3. The very faint band observed in this lane is likely due to marginal nonspecific binding of the anti‐4‐HNE antibody to the unmodified protein. The signal from 4‐HNE‐conjugated CYP4F11 confirms the presence of 4‐HNE adducts and the purity of the modified enzyme. To definitively confirm that enzyme activity is affected by 4‐HNE modification, we assessed the catalytic activity of nonmodified and 4‐HNE‐modified enzyme and observed significantly different Kcat values of 0.67 ± 0.12 and 0.02 ± 0.01, respectively (Figure 1D).

### 3.2 Raman and FTIR Spectroscopy Analysis of 4‐HNE‐Modified CYP4F11

**FIGURE 2:**
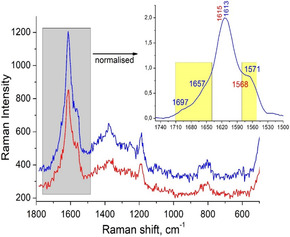
Changes in Raman spectra of 4‐HNE‐modified CYP4F11. Spectra of CYP4F11 (red line) and 4‐HNE‐conjugated CYP4F11 (blue line) are shown. In the inset, the spectra were normalized, and the wavenumber range 1750–1500 cm^−1^ is presented for 4‐HNE‐modified CYP4F11. The regions of Amide I and Amide II bands are highlighted in yellow, and the peaks for native CYP4F11 (in red) and 4‐HNE‐modified CYP4F11 (in blue) are indicated in cm^−1^. Measurements were performed in Dulbecco buffer (137 mM NaCl, 2.7 mM KCl, 8.1 mM Na_2_HPO_4_, and 1.5 mM KH_2_PO_4_, pH 7.4) in a quartz capillary tube with sample excitation at 244 nm.

**FIGURE 3:**
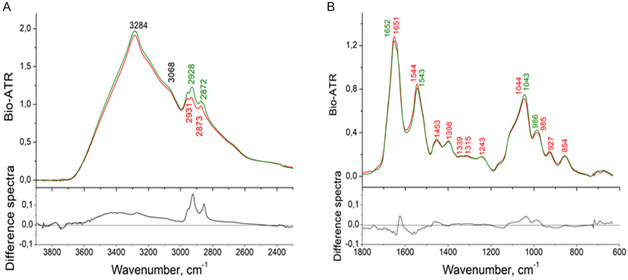
FTIR spectra of CYP4F11 (red line) and 4‐HNE‐modified CYP4F11 (green line) with the corresponding difference spectra (black line) at the bottom of the panels. The spectra were detected in Bio‐ATR (attenuated total reflection) units within the wavenumber ranges of 3800–2300 cm^−1^ (panel A) and 1800–600 cm^−1^ (panel B). The wavenumbers at which the characteristic peaks were observed are marked in the colour correspondent to line.

**FIGURE 4:**
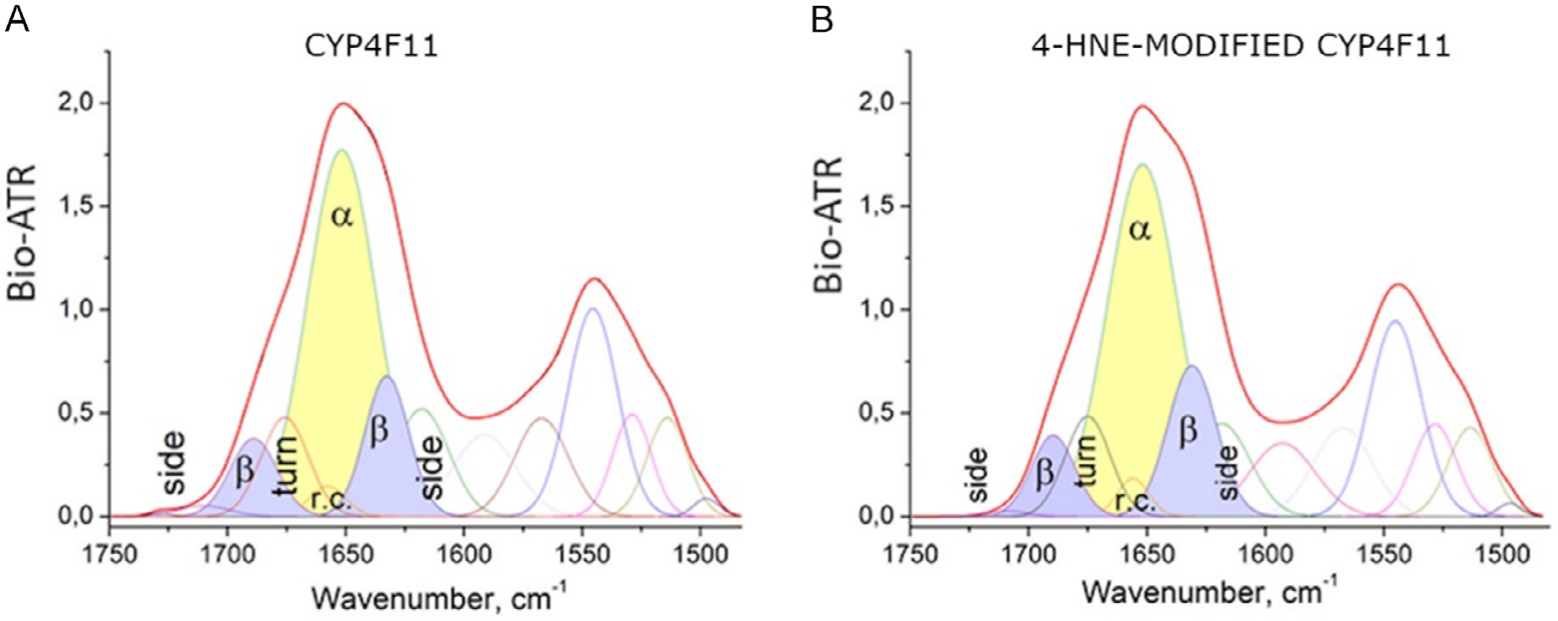
(A) FTIR spectra of CYP4F11 and (B) 4‐HNE‐modified CYP4F11 with their deconvolution. Spectra were analysed within the wavenumber range of 1750–1450 cm^−1^. The deconvolution of the CYP4F11 and 4‐HNE‐modified CYP4F11 signals (solid red line) into 13 components (multicoloured lines) was performed, showing both the experimental (solid red line) and predicted (solid black line, nearly covered by the solid red line) spectra. The components corresponding to the main elements of the secondary structure are labelled as “*α*” and filled in yellow for *α*‐helices, “*β*” in grey for *β*‐sheets, “turn” for turns, “r.c.” for random coils, and “side” for side groups. Deconvolution was conducted using PeakFit 4.0 FTIR analysis software.

Raman and FTIR measurements were conducted to investigate conformational changes in CYP4F11 following modification by 4‐HNE. Raman spectra for native and 4‐HNE‐modified CYP4F11 are shown in Figure 2, while FTIR spectra are presented in Figures 3 and 4. A summary of FTIR‐detected structural changes is provided in Table 1.

**TABLE 1:** Spectral characteristics of the amide i region and protein conformation assignments in 4‐HNE–modified CYP4F11 compared to nonmodified CYP4F11. Data derived from FTIR spectra.

Amide I band position and conformation contribution						
	CYP4F11			4‐HNE‐modified CYP4F11		
Secondary structure	Position, cm^−1^	Contribution, %	**Total, %**	Position, cm^−1^	Contribution, %	**Total, %**
***α*‐ helices**	1652	50.3 ± 0.1	**50.3 ± 0.1**	1652	50.1 ± 0.1	**50.1 ± 0.1**
***β*‐structure**: ‐*β*‐sheets	1633, 1689	19.8 ± 0.1	**29.2 ± 0.2**	1631, 1690	22.2 ± 0.1	31.4 ± 0.2*
‐Turns	1676	9.4 ± 0.1		1675	9.2 ± 0.1	
**Random conformation**: ‐Random coil	1658	2.2 ± 0.1	**20.4 ± 0.2**	1656	2.4 ± 0.1	18.5 ± 0.2*
‐Side groups	1618, 1710	18.2 ± 0.1		1619, 1609	16.1 ± 0.1	

*Note*: *β*‐sheets and turns are grouped under *β*‐structure, while random coil and side‐chain contributions are classified as random conformation. Contributions were determined based on peak positions characteristic of CYP4F11 and 4‐HNE–modified CYP4F11, measured independently across three separate preparations of CYP4F11. The standard error of mean is reported for *n* = 3 enzyme purifications. Significantly different values compared to unmodified CYP4F11 are reported as * for *p* < 0.01.

Both techniques revealed consistent alterations across the entire spectral range, with strong concordance between the Raman and FTIR results. In particular, the Raman spectrum of 4‐HNE‐modified CYP4F11 (Figure 2) showed changes in the intensity and distribution of C–H group vibrations likely involving both CH_2_ and CH_3_ groups, as well as a shift in the CH_3_/CH_2_ ratio. Significant shifts were also observed in the Amide I and Amide II bands, indicating modifications in the protein's secondary structure compared to the native form. In the Amide I and II region, shifts in band positions correlate with conformational changes in CYP4F11 (Figure 2, inset, yellow box on the right). In the C–H stretching region, both Raman and FTIR signal intensities increased (Figure 3A), which may reflect changes in the number and positioning of side chains in the modified protein. These structural alterations ultimately influence the overall protein conformation.

Second, in the FTIR spectrum of 4‐HNE‐modified CYP4F11, detectable changes—though not pronounced—were observed in both wavenumber regions: 3800–2300 cm^−1^ (Figure 3A) and 1800–600 cm^−1^ (Figure 3B). Although these changes are not easily visible to the naked eye in the graphs, spectral deconvolution of the 1750–1450 cm^−1^ range revealed a slight decrease in the *α*‐helix content, from 50.3 ± 0.1% to 50.1 ± 0.1%. (Figure 3, 4; Table 1). The combined fraction of *β*‐structures (*β*‐sheets plus *β*‐turns) increased significantly from 29.2 ± 0.2% to 31.4 ± 0.2% (Table 1), and the fraction of random coils increased significantly from 2.2 ± 0.1% to 2.4 ± 0.1% (Table 1). More detailed analysis of spectra deconvolution is reported in Supplemental Table S2.

### 3.3 Time‐Resolved Fluorescence Spectroscopy Analysis of 4‐HNE Modified CYP4F11

**FIGURE 5:**
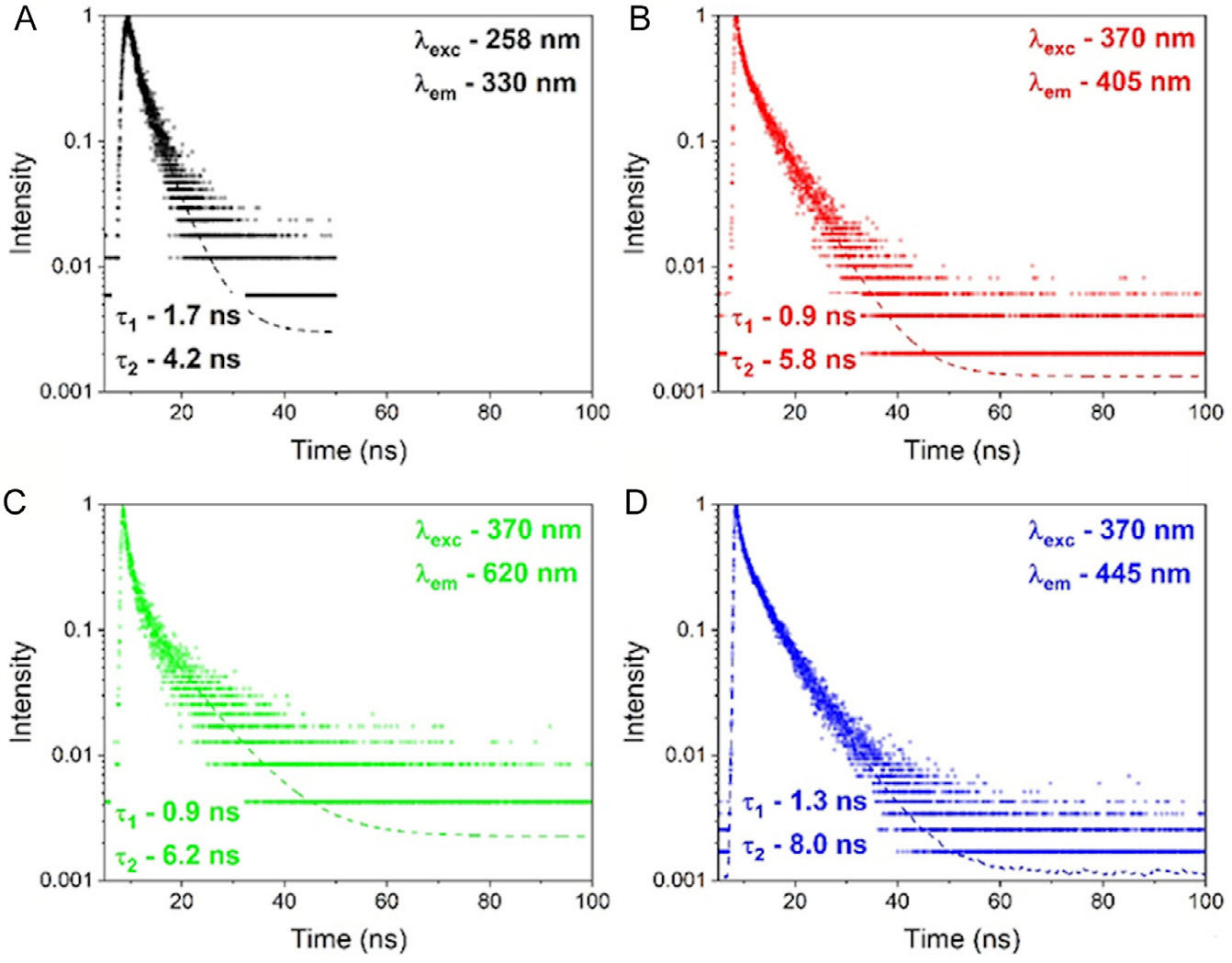
Luminescence decay of CYP4F11. Different excitation and emission wavelengths are reported: (A) *λ*_exc_ – 258 nm, *λ*_em_ – 330 nm, (B) *λ*_exc_ – 370 nm, *λ*_em_ – 405 nm, (C) *λ*_exc_ – 370 nm, *λ*_em_ – 620 nm, and (D) *λ*_exc_ – 370 nm, *λ*_em_ – 445 nm. The luminescence decay curves were deconvoluted by two‐exponential decay functions and the calculated lifetimes *τ*_1_ and *τ*_2_ were shown under each curves. The result of one typical experiment out of 3 is shown.

**FIGURE 6:**
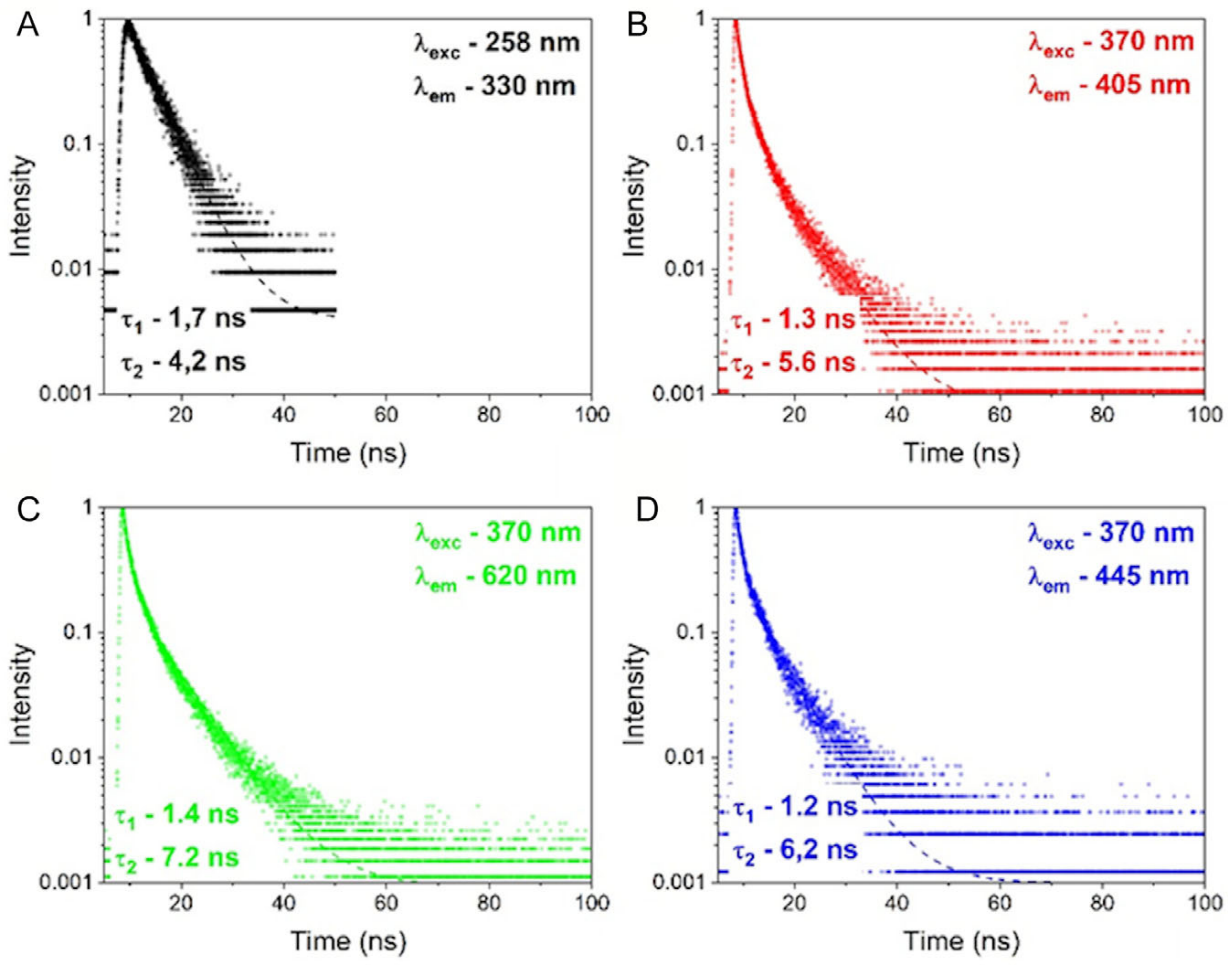
Luminescence decay of 4‐HNE‐modified CYP4F11. Different excitation and emission wavelengths are reported: (A) *λ*_exc_ – 258 nm, *λ*_em_ – 330 nm, (B) *λ*_exc_ – 370 nm, *λ*_em_ – 405 nm, (C) *λ*_exc_ – 370 nm, *λ*_em_ – 620 nm, and (D) *λ*_exc_ – 370 nm, *λ*_em_ – 445 nm. The luminescence decay curves were de‐convoluted by two‐exponential decay functions and the calculated lifetimes *τ*_1_ and *τ*_2_ were shown under each curves. The result of one typical experiment out of 3 is shown.

The luminescence decay curves of CYP4F11 were recorded using picosecond pulsed diode laser operating at 258 and 370 nm of excitation. The recorded decay curves shown nonexponential behaviour, indicating presence of the emission centres of the same origin in different sites. This indicates uniform distribution in the bond connections force between the samples. The recorded curves were fitted by two exponential function: $I=I_{0}+A_{1}e^{\left(\frac{-t}{\tau_{1}}\right)}+A_{2}e^{\left(\frac{-t}{\tau_{2}}\right)}$, where *I* is the luminescence intensity at time *t*, *I*_0_ is intensity value of initial background and *τ*_1_ and *τ*_2_ are fast and slow decay components, respectively, A1 and A2 are constants. The experimental luminescence decays and calculated luminescence lifetimes *τ*_1_ and *τ*_2_ are reported in Figures 5 and 6.

Differences in fluorescence lifetime were observed between native CYP4F11 (Figure 5) and 4‐HNE‐modified CYP4F11 (Figure 6) for two excitation wavelengths. We detected unchanged *τ*_1_ = 1.7 ns and *τ*_2_ = 4.2 ns for both unmodified (Figure 5A) and 4‐HNE‐modified CYP4F11 (Figure 6A) under excitation 258 nm and emission at 330 nm. The changes were observed at 370 nm excitation for *τ*_1_ and *τ*_2_: 0.9 ns and 5.8 ns in CYP4F11 versus 1.3 ns and 5.6 ns for 4‐HNE‐modified enzyme for 405 nm emission (Figure 5B and Figure 6B); 0.9 ns and 6.2 ns in CYP4F11 versus 1.4 ns and 7.2 ns for modified CYP4F11 for 620 nm emissions (Figure 5C and Figure 6C); 1.3 ns and 8.0 ns in CYP4F11 versus 1.2 and 6.2 ns for 4‐HNE‐modified CYP4F11 for 445 nm emission (Figure 5D and Figure 6D). Thus, a strong increase in the *τ*_1_ decay time by 44% and 56% with emissions at 405 nm and 620 nm, respectively, and an increase in the *τ*_2_ decay time by 16% with emission at 620 nm, were observed as a result of 4‐HNE conjugation, which means a significant change in protein structure with the appearance of distinct optically active centers. These variations may arise from multiple active centers within the same protein, such as oxygen vacancies, or from a single center exhibiting different properties under varying excitation and emission conditions. In our case, the observed changes are most likely attributable to structural differences and/or variations in binding forces between different regions of the protein, which influence its optical behaviour.

**FIGURE 7:**
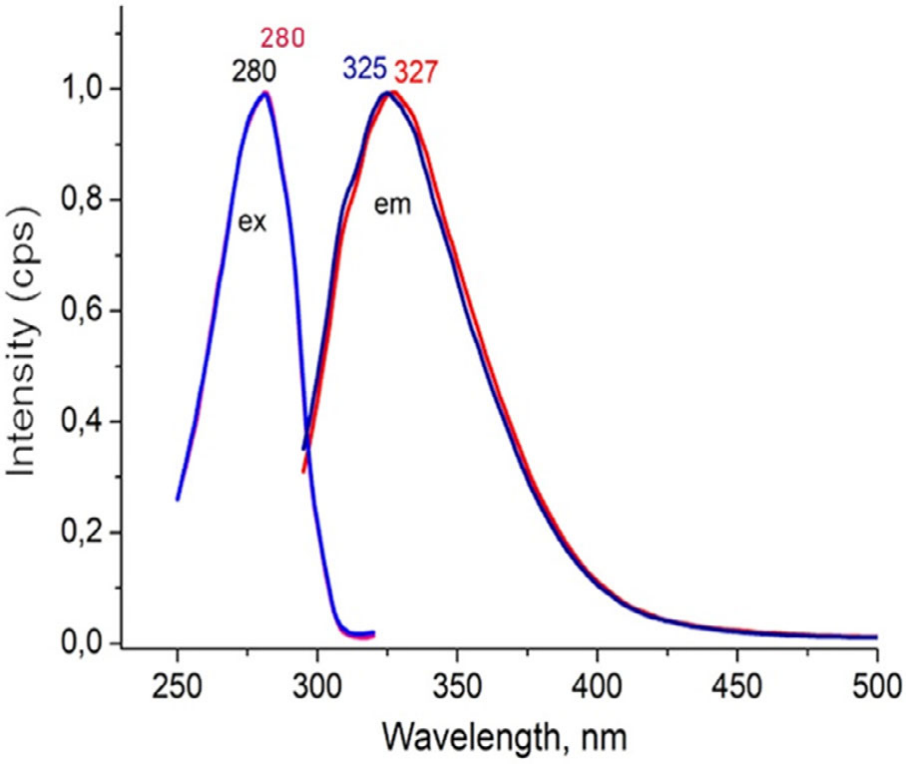
Fluorescence changes following CYP4F11 modification by 4‐HNE. Excitation (ex) and emission (em) spectra for native CYP4F11 are shown in red, for 4‐HNE‐modified CYP4F11 are displayed in light blue (excitation) and dark blue (emission). The spectral peaks are annotated above the graphs—red for native CYP4F11 and blue for the modified form. The data represent typical spectra obtained from three independent CYP4F11 preparations.

Additionally, excitation at a wavelength of 280 nm—corresponding to two nearly overlapping excitation spectra (Figure 7)—resulted in fluorescence emission at 327 nm for native CYP4F11 and at 325 nm for 4‐HNE‐modified CYP4F11, indicating a 2 nm shift in the fluorescence maximum. This shift is consistent with the luminescence decay data and supports the conclusion that 4‐HNE modification alters the structural and optical properties of the protein. The observed fluorescence changes suggest that 4‐HNE affects both the overall conformation of CYP4F11 and the binding interactions between different regions of the protein, leading to modifications in the behaviour of optically active centres.

### 3.4 DSC Analysis of 4‐HNE Modified CYP4F11

**FIGURE 8:**
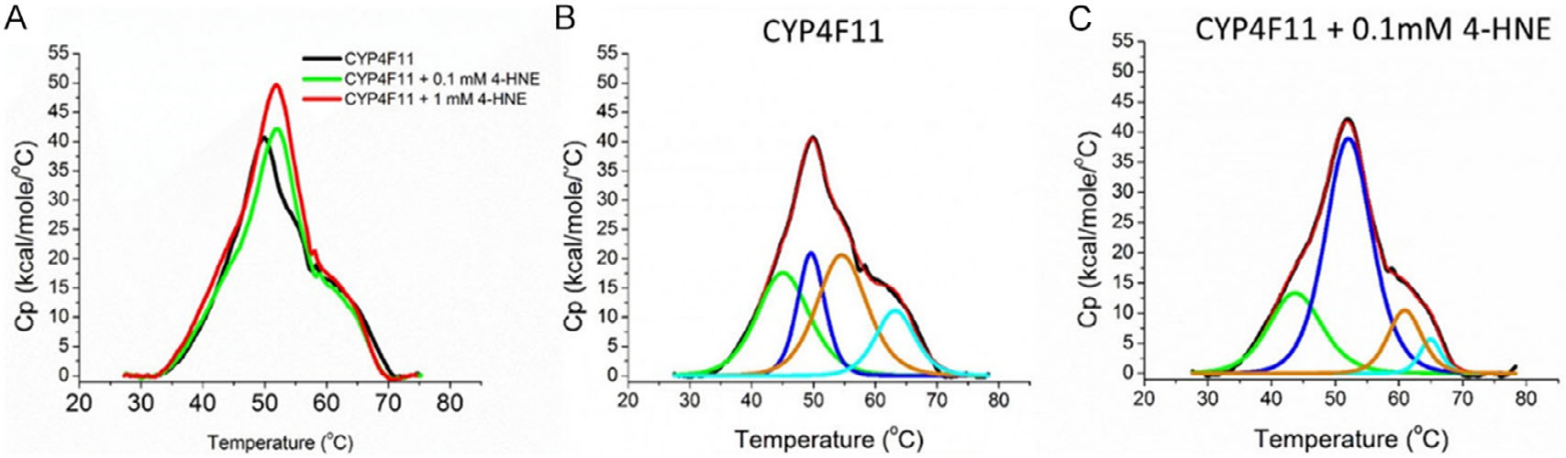
DSC thermograms of CYP4F11 and 4‐HNE‐conjugated CYP4F11 and their deconvolution. (A) Typical thermograms of CYP4F11 (black) and 4‐HNE‐conjugated CYP4F11 by 0.1 mM 4‐HNE (green) and 1 mM 4‐HNE (red). (B,C) Deconvolution of the thermograms obtained from CYP4F11 and CYP4F11 modified by 0.1 mM of 4‐HNE, respectively. Experimental thermograms are shown in black, fitting in red, the deconvoluted peaks 1, 2, 3, and 4 are in green, blue, orange, and light blue, respectively. Scan rate 60°C/h was applied to process the proteins concentrated 0.6 mg/mL in 50 mM KPi pH 7.4.

DSC was employed to monitor conformational changes in CYP4F11 induced by 4‐HNE modification. Three independent experiments were conducted for each condition: native CYP4F11, and CYP4F11 modified with 100 µM and 1 mM 4‐HNE, respectively. The thermogram corresponding to the 100 µM 4‐HNE‐modified sample, matching the concentration used in FTIR and Raman spectroscopy, is shown in Figure 8A. The thermogram for the sample modified with 1 mM 4‐HNE, used to assess dose‐dependent effects, is presented in Supplementary Figure S2. All DSC measurements were performed using a protein concentration of 0.6 mg/mL. Figure 8A shows that the addition of 4‐HNE results in the increase of the total enthalpy recorded for the thermogram and this effect is more pronounced when 1 mM 4‐HNE is used (Figure S2). Furthermore, deconvolution of the thermogram of CYP4F11, 100 µM 4‐HNE CYP4F11 or 1 mM 4‐HNE CYP4F11 always results in four peaks (Figures 8 and S2; Tables 2 and S1). The four peaks are all affected by the binding of 4‐HNE as clearly indicated by the increase in the *T*_m_ for peaks 2, 3 and 4, whereas peak 1 shows a slight decrease in the *T*_m_ (Tables 2 and S1). Both 0.1 mM and 1 mM 4‐HNE cause a dramatic change also in the enthalpy Δ*H* associated to each of the four peaks, indicating that a major modification is occurring in a dose‐dependent manner (Figures 8 and S2; Tables 2 and S1), mostly in the peak 3 (Tables 2 and S1, corresponding to the orange graph in the Figures 8B,C and S2) which has shifted dramatically, decreasing 3 times for 0.1 mM of 4‐HNE and almost 15 times for 1 mM 4‐HNE.

**TABLE 2:** The parameters of DSC thermogram deconvolution for CYP4F11 enzyme and 4‐HNE modified CYP4F11 (conjugated with 0.1 mM of 4‐HNE). The melting temperature *T*_m_ (°C) and enthalpy Δ*H* are reported for each peak. The standard error of mean is reported for *n* = 5 independent instrument measurements performed for *n* = 3 enzyme purifications.

	*T*_m_,°C				Δ*H*, kcal/mol/°C			
	PEAK 1	PEAK 2	PEAK 3	PEAK 4	PEAK 1	PEAK 2	PEAK 3	PEAK 4
**CYP4F11**	45.16 ± 0.43	49.64 ± 0.12	54.66 ± 0.48	63.26 ± 0.23	199.3 ± 19.4	123.8 ± 36.5	222.9 ± 49.7	95.4 ± 14.9
**4‐HNE‐modified CYP4F11**	43.81 ± 0.19*	52.14 ± 0.36*	60.96 ± 0.26*	64.99 ± 0.21*	148.7 ± 6.8*	379.6 ± 19.9*	73.9 ± 11.7*	25.3 ± 8.26*

Significantly different values compared to unmodified CYP4F11 are reported as * for *p* < 0.01.

### 3.5 Computational Prediction of Secondary Structure Changes

**FIGURE 9:**
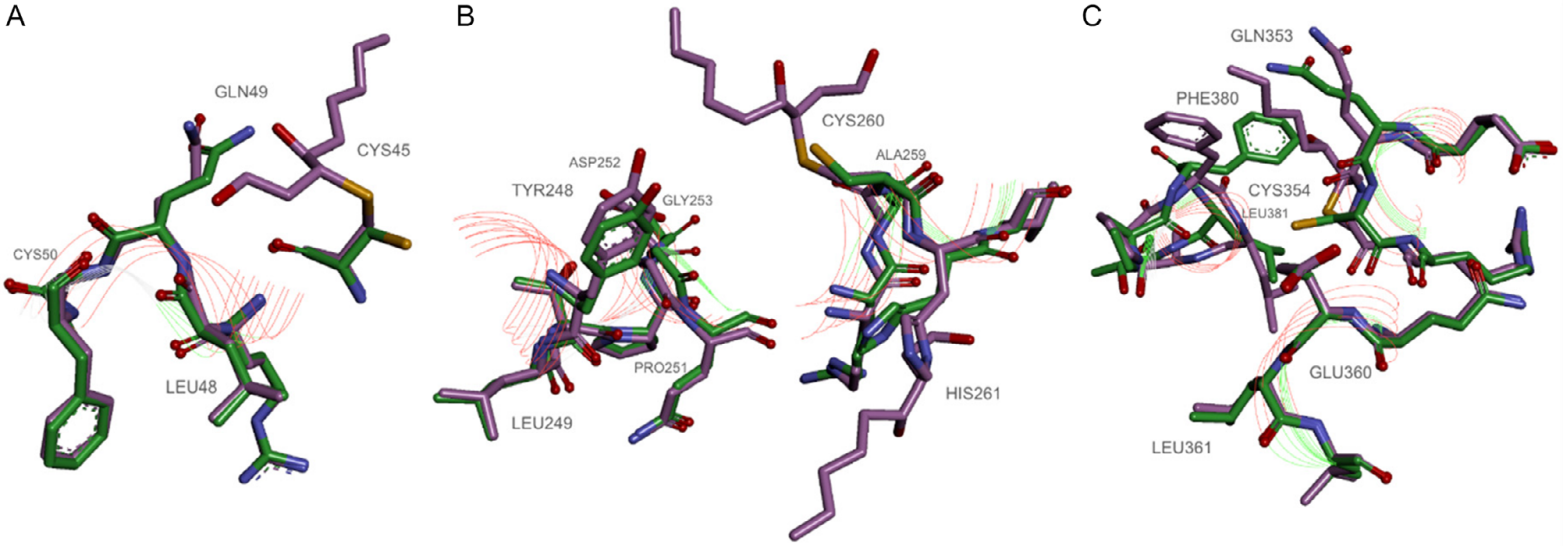
Predicted changes in the 4‐HNE modified structure of CYP4F11 (purple) relative to the original CYP4F11 structure (green). The 4‐HNE is shown in purple, as covalently bonded part of the protein, conjugated with CYS45 (panel A), CYS260 and HIS261 (B), and CYS354 (C). Red lines indicate helices, green lines represent coils. The modification at CYS45 induces changes in the secondary structure at positions 48–50 from helix to coil (panel A); the modifications at CYS260 and HIS261 induce changes in the secondary structure at positions 248–249, 251−253, and 259–260 from helix to coil (panel B); the modification at CYS354 induces changes in the secondary structure at positions 360–361 from helix to coil and at positions 353–354, 380−381 from coil to helix (panel C). The predicted structure of the CYP4F11 protein was generated by AlphaFold. Specific residues were manually modified by Michael addition with 4‐HNE. The modified structure was minimized using the UFF interaction model and RDKit v.2023.09.1 software.

To computationally identify structural changes in CYP4F11 induced by 4‐HNE conjugation, we utilized the open‐source cheminformatics tools RDKit and DSSP. Known amino acid modification sites previously identified by mass spectrometry [31] were manually incorporated into the model. Figure 9 illustrates examples of secondary structure alterations occurring near these modification sites. Notably, covalent attachment of the nine‐carbon 4‐HNE molecule at CYS45 leads to a transition in the secondary structure from helix to coil at positions 48–50 (Figure 9A), highlighting the local destabilizing effect of the modification. The modifications at CYS260 and HIS261 induce changes in the secondary structure at positions 248–249, 251−253, and 259–260 from helix to coil (Figure 9B). The modification at CYS354 induces changes in the secondary structure at positions 360–361 from helix to coil and at positions 353–354, 380−381 from coil to helix (Figure 9C).

A summary of all secondary structure changes is provided in Table 3. Computational analysis revealed a 2.5% decrease in *α*‐helical content (from 53.6% to 51.1%), a 3.3% increase in *β*‐structures (from 29.2% to 32.5%), and a 0.9% decrease in coil regions (from 17.1% to 16.2%). These computationally predicted changes closely mirror the experimental observations obtained by FTIR spectroscopy (Table 1), supporting the conclusion that 4‐HNE conjugation induces measurable alterations in the secondary structure of CYP4F11.

**TABLE 3:** Computational modelling of CYP4F11 and 4‐HNE‐modified CYP4F11 conformations using the DSSP program.

Secondary structure with definition	CYP4F11	4‐HNE modified CYP4F11
**Helices** – 4‐turn helices (*α*‐helices) with minimum length 4 residues – 3‐turn helices (3_10_ helices) with minimum length 3 residues	53.6%	51.1%
***β*‐ structure** – Hydrogen bonded turns (3, 4, or 5 turn) – Extended strands in parallel and/or anti‐parallel *β*‐sheet conformations – Residues in isolated *β*‐bridges (single pair *β*‐sheets hydrogen bond formation) – Bends (the only nonhydrogen‐bond based assignment, high curvature)	29.2%	32.5%
**Random conformation** – Coils (residues which are not in any of other conformations) – Other undefined residues	17.1%	16.2%

## 4 Discussion

Oxidative stress, accompanied by lipid peroxidation and ferroptosis, is a key feature of numerous diseases, including chronic inflammatory and infectious conditions such as malaria [9, 73, 74, 75, 76, 77, 78, 79]. Certain lipid peroxidation products can conjugate with exposed proteins, often impairing their functionality [8, 30, 80, 81]. Among the terminal products of lipid peroxidation, 4‐HNE is capable of forming Michael adducts and Schiff bases with proteins and nucleic acids [4, 82]. Several cytochrome P450 enzymes, including CYP1A1, CYP1A2, CYP2E1, and CYP4F11, have been shown to undergo modification by 4‐HNE [31, 83, 84]. CYP4F11 plays a role in lipid metabolism, specifically catalyzing the *ω*‐hydroxylation of arachidonic acid and other substrates such as 12‐HETE and 15‐HETE [31, 45, 48, 85, 86, 87]. After conjugation with 4‐HNE, six amino acids were identified as modified by mass spectrometry: C45, C260, H261, H347, C354, and K451 [31]. C260 and H261 are in the substrate recognition site of CYP4F11 [88], and K451 is a ubiquitination site on CYP4F11 [89]. While the exact impact of these sites is unknown, 4‐HNE‐modified CYP4F11 showed strong inhibition of enzymatic activity [31], with the inhibition mechanism yet to be studied and with great potential of this knowledge for application in other fields where CYP4F enzymes have a role [47] or 4‐HNE–protein modifications have a role [8, 14, 24].

In this study, we analysed the structural changes in CYP4F11 modified by 4‐HNE using DSC, FTIR, and Raman spectroscopy. The concentration of 4‐HNE was quantified by HPLC, with a detection limit of 0.25 µM (Figure 1A), corresponding roughly to the detection limit described by others for HPLC [25]. In further studies, we plan to enhance the detection limit for 4‐HNE in our experiments by employing sample derivatization and HPLC‐mass spectrometry (HPLC‐MS) techniques. The kinetics of 4‐HNE binding to CYP4F11 were measured (Figure 1). During incubation with CYP4F11, the free 4‐HNE concentration decreased in the medium (Figure 1B), indicating progressive binding to CYP4F11. The efficient conjugation was additionally confirmed by antibodies specific to 4‐HNE‐adducts via WB (Figure 1C). We observed a strong positive signal from the modified CYP4F11 and no signal from the nonmodified enzyme (Figure 1C). To confirm that 4‐HNE modification significantly affects enzyme activity, we measured the catalytic activity of nonmodified and 4‐HNE–modified enzyme and found markedly different Kcat values (Figure 1D), meaning that 4‐HNE conjugation almost completely abolishes catalytic function. In the future, we plan to study a wide spectrum of conditions for 4‐HNE binding, aiming to better reflect possible in vivo environments, such as enzyme microenvironments in terms of acidity, osmolarity, or lipid content. Mass spectrometry analysis of 4‐HNE binding sites under these different conditions will also be performed to deepen our understanding.

Based on spectroscopic data and computational modelling of the 4‐HNE‐modified CYP4F11, we identified some structural changes in the *α*‐helices, *β*‐phase (*β*‐sheets and turns), and disordered phases or random coils compared to the unmodified CYP4F11. To underline, the spectral shifts are due to covalently linked 4‐HNE, which modifies the protein structure, reflected in the spectra, and additionally, 4‐HNE itself may contribute to the shift.

Several molecular modeling software programs, including open‐source PyMOL, BIOVIA Discovery Studio, and DSSP, which we also attempted to use to analyze the experimental data, showed slight differences in the quantification of conformational states. Nevertheless, the results from all the software were highly similar, providing consistent information about the structural changes induced by 4‐HNE. Thus, according to our calculations, the helical content is decreased by 2.5%, the *β*‐structures (sheets and turns) are increased by 3.3%, and the random configuration is decreased by 0.9% (Table 3). Experimental data after fitting showed *α*‐helices decrease by 0.23%, *β*‐structures increased by 2.1%, and the random configurations decreased by 1.89% (Table 1). Although not exactly identical between theoretical and experimental approaches, the changes in the secondary structure elements were in the same direction and of the same order.

Another method, DSC, further supports and confirms the presence of structural alterations in CYP4F11 resulting from exposure to 4‐HNE. While the overall unfolding of the protein is not strongly affected, it is significantly influenced by the binding of 4‐HNE. The melting temperature (*T*_m_) of unmodified CYP4F11 was 49.85 ± 0.15°C, whereas CYP4F11 modified with 100 µM 4‐HNE showed a *T*_m_ of 52.75 ± 0.41°C, and modification with 1 mM 4‐HNE resulted in a *T*_m_ of 52.12 ± 0.31°C (Figure 8A). These results suggest that covalent modification by 4‐HNE induces local structural reorganizations within the protein's secondary structure. These changes are evident in the thermograms after deconvolution, appearing as shifts in the *T*_m_ of all peaks in the protein solution, with an overall trend toward stabilization, except for peak 1, which shows a decrease in *T*_m_ of about 2°C (Table 2). The most significant structural change is detected in the dramatic increase in terms of enthalpy for peak 2, where a gain of 256 kcal/mol can be observed (Table 2) and mush more strong for high 4‐HNE concentration (Table S1). Modification with 4‐HNE may stabilize specific regions of the CYP4F11 protein, including enzymatically important domains, thereby making unfolding more energetically demanding. We propose that covalent binding of 4‐HNE induces both local and global conformational changes, which affect the thermodynamic profile of the protein and involve rearrangements in its secondary structure. Overall, the changes are consistent with ligand‐induced modifications previously reported for other proteins [38, 68, 69].

To address the limitations and future perspectives of the computational part of this study, we considered the local minimization of AlphaFold‐predicted structures for both modified and unmodified proteins. While this approach provides a useful comparative framework, it does not guarantee an accurate representation of the native equilibrium conformation. Therefore, the structural interpretations should be considered with caution. The MD simulations could rectify the above mentioned limitation, however, performing MD of a protein structure with specific residue modifications is prohibitively hard to set up and is out of the scope of this work. Therefore, in the next iteration of modeling the modified protein structure, our main effort will be devoted to MD simulations and to leveraging the continuously expanding capabilities of AlphaFold. Obviously, the most effective way to address our research questions would be through a comparative analysis of the X‐ray structures of both the unmodified and 4‐HNE‐modified CYP4F11. This approach presents a promising direction for further development of the study.

Altogether, by applying biochemical and biophysical methods, we observed changes in 4‐HNE‐modified CYP4F11 that lead to structural alterations of the enzyme.

## 5 Conclusions

In conclusion, our study provides new insights into the impact of 4‐HNE modification on CYP4F11, revealing specific alterations in its secondary structure and enzymatic activity. These structural changes can significantly affect lipid metabolism, particularly under conditions of elevated oxidative stress and lipid peroxidation, such as those observed in malaria and acute or chronic inflammatory diseases. While current modeling approaches offer a useful comparative framework, future efforts will focus on MD simulations and the integration of evolving AlphaFold capabilities to achieve more accurate representations of modified protein conformations. These findings lay the groundwork for broader applications in understanding lipid peroxidation‐driven protein modifications across diverse biological contexts.

## 6 Statistical Analysis

The values from at least three independent replicates are presented in histograms (means ± standard errors, SE). Statistical significance was calculated by Mann–Whitney test (PASW Statistics 18, IBM SPSS, Armonk, NY, USA), and *p* values below 0.01 were considered statistically significant.

## Supporting Information

Additional supporting information can be found online in the Supporting Information section. **Supporting Fig. S1:** Spectral analysis of heterologously expressed and purified CYP4F11. CYP4F11 expression and purification were followed by a CO difference spectrum assay. (A) Absorbance spectra of the oxidized (blue line), reduced (red line), and reduced CO‐bound (green line) forms of CYP4F11. (B) Difference spectrum comparing CO‐bound reduced and reduced forms of CYP4F11. **Supporting Fig. S2:** Differential scanning calorimetry (DSC) thermogram of 4‐HNE‐conjugated CYP4F11 and its deconvolution. Deconvolution of the thermogram obtained from CYP4F11 modified by 1 mM of 4‐HNE. Experimental thermogram is shown in black, the fitting is in red and the deconvoluted peaks 1, 2, 3, 4 are in green, blue, orange and light blue respectively. Scan rate 60°C/h was applied to process the proteins concentrated 10 μM in 50 mM KPi pH 7.4. **Supporting Table S1:** The parameters of DSC thermogram deconvolution for CYP4F11 enzyme modified with 1 mM of 4‐HNE. Peaks 1, 2, 3, 4 are represented in Figure S1. The standard error of mean is not reported in Table S1 because it is below 1.5% of each of the reported data. **Supporting Table S2:** Data from fitting of Amid I band of FTIR spectra for CYP4F11 and 4‐HNE‐modified CYP4F11 proteins.

## Funding

The work was supported by University of Turin (Grant SKOO_CONTR_FIN_22_01 “Support actions in favour of Ukrainian scholars and students”).

## Conflicts of Interest

The authors declare no conflicts of interest.

## Data Sharing Statement

The data supporting the findings of this study are available within the article and its Supplementary Materials.

## Supporting information

Supplementary Material

## Data Availability

The original contributions presented in the study are included in the article/supplementary material, further inquiries can be directed to the corresponding authors.
