# Supplementary material for: Structural Impact of 4‐Hydroxynonenal Modification on Human Cytochrome CYP4F11
Source: ChemMedChem. 2026 Jan 31;21(2):e202500935. doi: 10.1002/cmdc.202500935 (PMC12860540; doi:10.1002/cmdc.202500935)
Supplement: Supplementary file 1 — Supplementary Material [file CMDC-21-e202500935-s001.pdf]

**Supplementary Materials** to the original paper Gnatyuk at al., "Structural impact of 4-hydroxynonenal modification on human cytochrome P450 4F11".

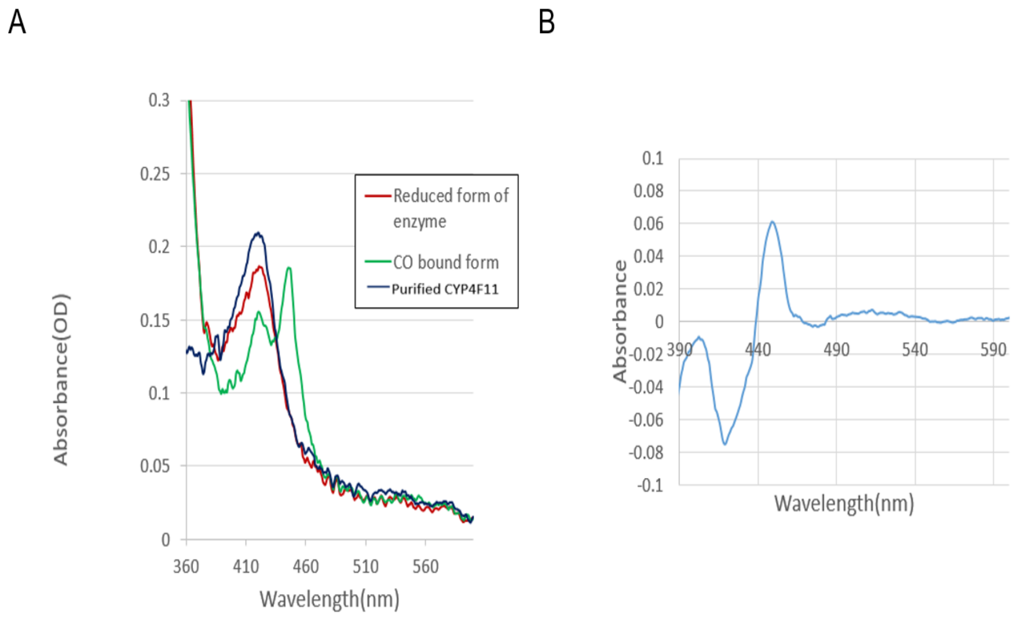


Figure S1. Spectral analysis of heterologously expressed and purified CYP4F11. CYP4F11 expression and purification were followed by a CO difference spectrum assay. (A) Absorbance spectra of the oxidized (blue line), reduced (red line), and reduced CO-bound (green line) forms of CYP4F11. (B) Difference spectrum comparing CO-bound reduced and reduced forms of CYP4F11.

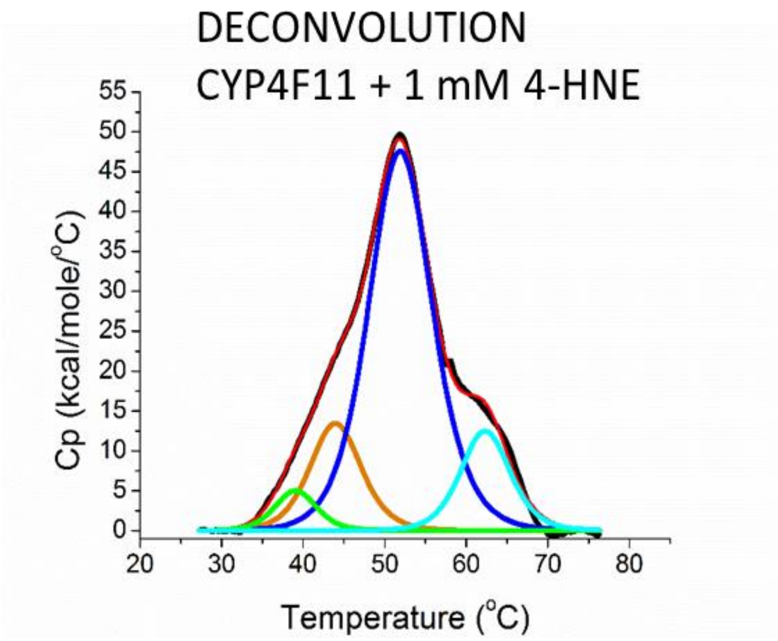


Figure S2. Differential scanning calorimetry (DSC) thermogram of 4-HNE-conjugated CYP4F11 and its deconvolution. Deconvolution of the thermogram obtained from CYP4F11 modified by 1 mM of 4-HNE. Experimental thermogram is shown in black, the fitting is in red and the deconvoluted peaks 1, 2, 3, 4 are in green, blue, orange and light blue respectively. Scan rate 60 °C/h was applied to process the proteins concentrated 10 µM in 50 mM KPi pH 7.4.

Table S1. The parameters of DSC thermogram deconvolution for CYP4F11 enzyme modified with 1 mM of 4-HNE. Peaks 1, 2, 3, 4 are represented in Figure S1. The standard error of mean is not reported in Table S1 because it is below 1.5% of each of the reported data.

| | **$T_m$ Melting T/°C** | | | | **ΔH kcal/mol/°C** | | | |
|---|---|---|---|---|---|---|---|---|
| | PEAK 1 | PEAK 2 | PEAK 3 | PEAK 4 | PEAK 1 | PEAK 2 | PEAK 3 | PEAK 4 |

| | | | | | | | | |
|---|---|---|---|---|---|---|---|---|
| **CYP4F11 + 1 mM 4-HNE** | 44.0±0.39 | 51.97±0.88 | 39.08±0.84 | 62.36±0.94 | 111.9±3.7 | 492.6±8.2 | 33.9±2.3 | 100.9±4.2 |

Table S2. Data from fitting of Amid I band of FTIR spectra for CYP4F11 and 4-HNE-modified CYP4F11 proteins

**CYP4F11**

| **Area** | **Amplitude** | **Center** | **Width** |
|---|---|---|---|
| 13.22 | 0.40 | 1591 | 31.36 |
| 14.91 | 0.52 | 1618 | 26.83 |
| 15.81 | 0.68 | 1633 | 21.82 |
| 64.07 | 1.78 | 1652 | 33.86 |
| 2.81 | 0.15 | 1658 | 17.85 |
| 11.99 | 0.48 | 1676 | 23.41 |
| 9.46 | 0.38 | 1689 | 23.51 |
| 1.45 | 0.05 | 1710 | 25.93 |
| 0.20 | 0.02 | 1728 | 10.59 |

**4-HNE modified CYP4F11**

| **Area** | **Amplitude** | **Center** | **Width** |
|---|---|---|---|

| | | | |
|---|---|---|---|
| 12.32 | 0.36 | 1593 | 32.59 |
| 13.59 | 0.45 | 1618 | 28.33 |
| 18.89 | 0.73 | 1631 | 24.24 |
| 63.23 | 1.71 | 1652 | 34.81 |
| 3.06 | 0.19 | 1656 | 15.51 |
| 11.58 | 0.49 | 1675 | 22.39 |
| 9.14 | 0.39 | 1690 | 21.76 |
| 0.56 | 0.03 | 1709 | 19.88 |
